# Sevoflurane usage and fresh gas flows in Maquet anaesthetic machines at an academic hospital

**DOI:** 10.4102/jcmsa.v3i1.170

**Published:** 2025-06-13

**Authors:** Aobakwe R. Setlhare, Kylesh D. Pegu, Mathabe Sehlapelo

**Affiliations:** 1 Department of Anaesthesiology, Faculty of Health Science, University of the Witwatersrand, Johannesburg, South Africa; 2 Anaesthesiologist in Private Practice, Johannesburg, South Africa

**Keywords:** sevoflurane, low-flow anaesthesia, cost, cost-containment, Maquet Flow-i^®^, environment, South Africa

## Abstract

**Background:**

Low-flow anaesthesia (LFA) is crucial in combating rising healthcare costs and the global threat of climate change. This study analysed the conduct of inhalational anaesthesia at a Johannesburg Academic Hospital to determine fresh gas flows (FGF) and liquid agent consumption (LAC) at various stages of anaesthesia.

**Methods:**

A prospective, contextual research design was followed. Purposive sampling method was used in 10 theatres equipped with Maquet Flow-i® anaesthetic machines. Calculated LAC values were compared to those measured by the anaesthetic machines.

**Results:**

The average FGF during induction, maintenance and time-weighted case average were 7.07 L/min, 1.41 L/min and 1.73 L/min, respectively. The average end-tidal sevoflurane concentration during maintenance was 2.40%. The calculated average LAC for induction, maintenance and total case were 7.74 mL, 28.01 mL and 36.84 mL, respectively, while the hourly LAC was 16.71 mL/h. The calculated case average LAC overestimated the measured values by 4.14 ± 4.86 mL (12.98%), with 98.5% of values being within ± 1.96 standard deviation (s.d.). Despite its brevity, the induction phase accounted for 21% of the calculated LAC. The calculated liquid agent expenditure over time was ZAR54.32 ± 23.55/h. Case average FGF had a very high positive correlation with the calculated cost of sevoflurane, *r* = 0.86, *p* < 0.001.

**Conclusion:**

This study demonstrated that the prevailing use of medium-flow anaesthesia among anaesthetists at our institution resulted in significant sevoflurane wastage, increased expenditure and environmental pollution.

**Contribution:**

The study provides insight into anaesthesia practices at an academic hospital. It highlights the need to implement policies to standardise LFA as a cost-saving and environmentally friendly strategy.

## Introduction

The cost of providing healthcare services, facilities and technologies continues to rise while resources remain finite.^1,2,3^ This is evident in developing countries like South Africa, where a large demand-supply gap exists for healthcare services.^4^ As a result, it is imperative that hospitals implement cost-containment measures without compromising patient care.

One measure of potential cost reduction involves the usage of volatile anaesthetics (VA). According to Rinehardt and Sivarajan,^5^ in the United States, anaesthesia medications account for 10% – 13% of pharmacy budgets. With the adoption of voluntary protocols to reduce wastage, they further note a potential to yield savings of $350 – $750 million annually. It is estimated that VA accounts for 20% of the total anaesthetic drug cost.^6^ Therefore, implementing cost-containment and awareness programmes would be advantageous because of the low cost-consciousness among healthcare providers.^7,8^

Low-flow anaesthesia (LFA) has demonstrated significant potential for cost reduction and decreased environmental impact associated with VA.^9,10,11^ Baker^12^ classified fresh gas flows (FGF) in litres per minute (L/min) into six categories: metabolic flow (0.25), minimal flow (0.25–0.5), low-flow (0.5–1.0), medium flow (1–2), high flow (2–4) and very high flow (> 4). While the overall contribution of VA to greenhouse emissions is relatively minor, it is essential to minimise their release into the environment.^13^ Advancements in anaesthesia machines and carbon dioxide absorbents have facilitated the safe and efficient implementation of LFA in clinical practice.^14,15^

The key determinants of overall FGF and liquid agent consumption (LAC) under the anaesthetist’s direct control are induction FGF, induction duration and maintenance FGF.^16^ Although very important in overall LAC, the maintenance duration is beyond the control of the anaesthetist. Numerous international studies that discuss various costs of anaesthesia care have been published.^1,5,17^ However, to the best of our knowledge, the only published South African study that addressed the cost of inhalational anaesthesia was by Ryksen and Diedericks in 2014.^18^

This study aimed to prospectively analyse the conduct of inhalational anaesthesia at Charlotte Maxeke Johannesburg Academic Hospital (CMJAH) to determine FGF and the LAC at different stages of anaesthesia.

## Methods

A prospective, contextual research design was conducted from 15 May 2023 to 26 May 2023. The study population consisted of data logs from Maquet Flow-i^®^ anaesthetic machines (AM) at CMJAH. This model was selected as it was the sole anaesthetic machine at our institution capable of exporting data logs directly to a Universal Serial Bus (USB) drive without the need for additional proprietary software or licensing.

Purposive sampling method was used. To compare our cohort’s continuous mean primary outcome against a literature-derived standard, we selected the study by Nair et al.^3^ for comparative analysis. They reported a baseline FGF rate of 2.27 ± 1.05 L/min and our anticipated mean was 1.9 L/min. Consequently, a minimum sample size of 105 cases was required to achieve an alpha of 0.05 and a power of 95%.

All data from elective and emergency general anaesthetic cases conducted with a Maquet Flow-i^®^ AM throughout the study period, including weekends, were collected. Cases that used closed circuit ventilation with either a laryngeal mask airway or endotracheal tube, lasted longer than 45 min and used sevoflurane as the sole VA, were included in the study. In all cases where an auxiliary common gas outlet (ACGO) was used, maintenance minimum alveolar concentration (MAC) of less than 0.7 or AM with a leak greater than 150 mL/min was excluded from the study.

Data were collected at the end of each elective theatre list and 12 hourly for emergency theatres by inserting a USB drive into the rear USB port of the anaesthetic machine by the primary researcher. The primary researcher was not present during any of the cases. The menu button was selected. The ‘save all data logs and trends to USB’ were selected using the touch interface. The saved data were then consolidated into a single Microsoft Excel^®^ spreadsheet. Cases were identified by continuous data logs recorded at 1-min intervals. Any data entry break longer than 10 min was considered the end of a case, with subsequent entries being the start of another. There were no missing data. Cases that met the inclusion criteria were identified and analysed further.

The phases of anaesthesia were defined as follows: the induction phase, where FGF was greater than or equal to 5 L/min; the transition phase, where FGF was greater than or equal to 2 L/min but less than 5 L/min and the maintenance phase, where FGF was less than 2 L/min and ended when the vaporiser was turned off.^16^ Movement between phases was unidirectional, which meant that once the maintenance phase was entered, all subsequent adjustments formed part of maintenance regardless of the FGF. The ambient theatre temperatures and barometric pressures were recorded at 11:00 and 14:00 daily. This process was repeated in all 10 theatres.

The first step was to determine the saturated gas volume using the formula below^19^:

$$Saturated\;Gas\;Volume\;(mL/mL)=\frac{specific\;weight^{1}.Avogadro's\;gas\;constant^{2}.{\left(273+temperature\right)}^{3}}{molecular\;weight^{4}.273}$$ [Eqn 1]

- Specific weight of sevoflurane = 1.53 grams per millilitre (g/mL)
- Avogadro’s gas constant states that at a standard atmospheric pressure of 760 mmHg (at sea level) and at a temperature of 0 °C = 273 Kelvin (°K) one mole of any gas consists of 6.023 × 10^23^ molecules, which in turn occupy a volume of 22 400 mL. This is the same for all gases, VA included, at standard temperature, standard pressure and dry conditions
- Temperature of vaporiser = room temperature – 2 °C due to loss of energy during evaporation
- Molecular weight of sevoflurane = 200.

The calculations accounted for the elevation of Johannesburg, 1760 m above sea level,^20^ and daily barometric pressures when calculating Avogadro’s gas constant. Daily ambient theatre temperatures were used in the formula. The saturated gas volume obtained was then used to calculate the fluid volatile agent consumed per time segment as shown below^19^:

$$fluid\;volatile\;agent\;(mL)=\frac{FG\;flow\;\frac{mL}{\min}.VA\;conc.(Vol\%).Anaesthesia\;duration\;(\min)}{Saturated\;gas\;volume\;\frac{mL}{mL}.100(Vol\%)}$$ [Eqn 2]

Abbreviations:

- FG – fresh gas
- VA conc. – volatile anaesthetic concentration
- Vol – volume
- mL/min – millilitres per min
- min – minute

The calculated volumes per time segment were then summed up to get the total volumes per phase of anaesthesia and for the entire duration of the anaesthetic. The vaporisers in the Maquet Flow-i^®^ use an electronic injection system whereby measured quantities of liquid agent evaporate to produce known quantities of anaesthetic vapour.^21^ Therefore, to verify the accuracy of calculated volumes, they were compared to the measured values recorded by the AM. The procurement cost of sevoflurane as per government tender in May 2023 was used to determine calculated costs.

Data were exported to IBM SPSS 28^®^ for analysis. The majority of the results were descriptive results. Pearson correlation and regression analysis were used to explore the relationship between calculated and measured values. Overall FGF was calculated using time-weighted means to enable comparison with other published literature. Similar to a study by Kennedy et al.,^16^ the underlying data may not be normally distributed; however, this value represented the average FGF and, therefore LAC. The flow rates at different stages of anaesthesia were described with the median, interquartile range and 85th centile to quantify outliers. The relationship between LAC and case average FGF was also explored using linear regression analysis.

### Ethical considerations

Ethical clearance to conduct this study was obtained from the University of the Witwatersrand Human Research Ethics Committee (Medical) (No. M230147) and other relevant authorities.

## Results

**FIGURE 1:**
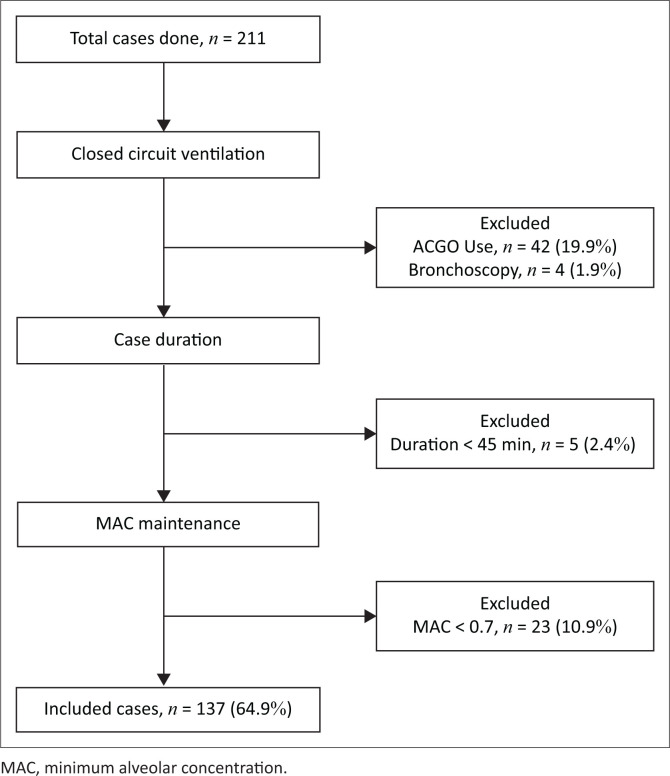
Case selection flow diagram.

The study recruitment flow diagram in Figure 1 was used for case selection. In total, 211 sevoflurane general anaesthesia cases were performed in 10 operating theatres using Maquet Flow-i^®^ AM.

Inhalational agent concentration during the maintenance period was found to be most representative of the depth of anaesthesia maintained in our study. The distribution of average maintenance end-tidal sevoflurane (ETS) across all theatres is shown in Table 1. The paediatric theatres 1 and 2 demonstrated significantly higher average ETS (2.94% and 2.99%, respectively) compared to other theatres (*p* < 0.001), except theatre 6 (2.57%) which accommodates both paediatric and adult cases (*p* = 0.500 and *p* = 0.061, respectively).

**TABLE 1:** Pattern of fresh gas flows rates at various phases of anaesthesia, average MAC and Maintenance End-tidal sevoflurane.

Descriptive statistics	Induction FGF (L/min)	Induction duration (min)	Maintenance FGF (L/min)	Case average FGF (L/min)	Average MAC	Average maintenance ETS (%)
Mean	7.07	6.69	1.41	1.79	0.90	2.40
s.d.	1.83	5.73	0.7	0.73	0.1	0.42
Minimum	0.00	0.00	0.68	0.93	0.71	1.52
25th centile	6.00	3.00	1.03	1.27	0.82	2.11
50th centile	6.52	6.00	1.17	1.61	0.90	2.38
75th centile	8.00	9.00	1.55	2.08	0.96	2.62
85th centile	9.00	11.00	1.91	2.30	1.00	2.80
Maximum	12.90	39.00	6.23	6.21	1.24	3.84

s.d., tandard deviation; ETS, end-tidal sevoflurane; FGF, fresh gas flow; MAC, minimum alveolar concentration.

These ETS concentrations corresponded with an average maintenance MAC value of 0.90 ± 0.10 across all theatres. The one-way analyses of variance (ANOVA) test showed a significant difference in MAC across theatres, F (9, 127) = 1.97, *p* = 0.048. Tukey’s post-hoc test was used to compare the groups in pairs to determine which was significantly different. Despite the significant difference in the ANOVA, no pairwise group comparison was significant in Tukey’s post-hoc test; all *p*-values were greater than 0.05.

The distribution of average FGF for induction, maintenance and overall case are shown in Table 1. Sevoflurane was administered for a total of 19 969 min. This time was distributed as follows: induction (916 min, 4.6%), transition (328 min, 1.6%) and maintenance (18 725 min, 93.8%). The average transition phase duration was 2 min per case. Given the relatively short duration of the transition phase (1.6% of the total time), further analysis of this phase was deemed unnecessary. This period was however included when calculating case average FGF for each case.

**FIGURE 2:**
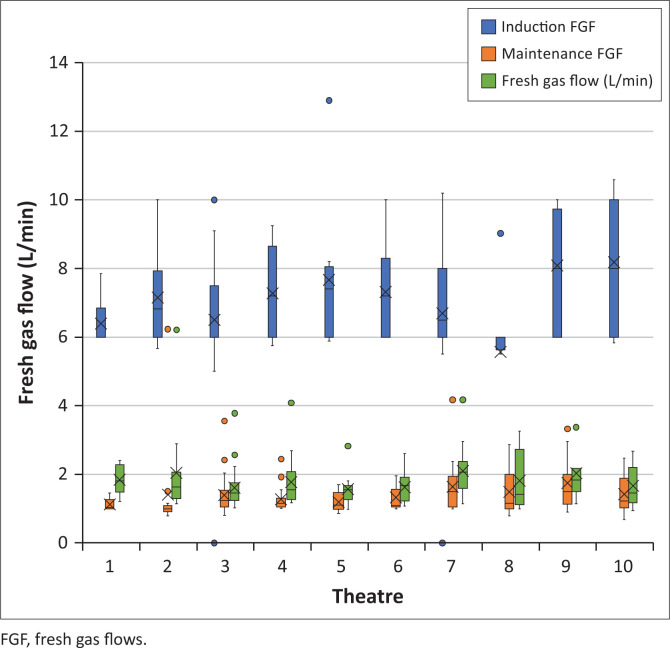
Theatre fresh gas flows per time segment.

The results for average FGF at induction, maintenance and overall case average FGF are depicted in Figure 2. Average FGFs were pooled because the one-way ANOVA test showed no significant differences in case average FGF across theatres F (9, 127) = 1.040, *p* = 0.412. The case average FGF, 1.79 ± 0.73 L/min, summarises the mean of the individual cases. This value is independent of the individual case duration and therefore, differs from the time-weighted pooled mean of 1.73 L/min. Paired *t*-test analysis showed that case average FGF was significantly higher than maintenance FGF, *p* < 0.001.

**FIGURE 3:**
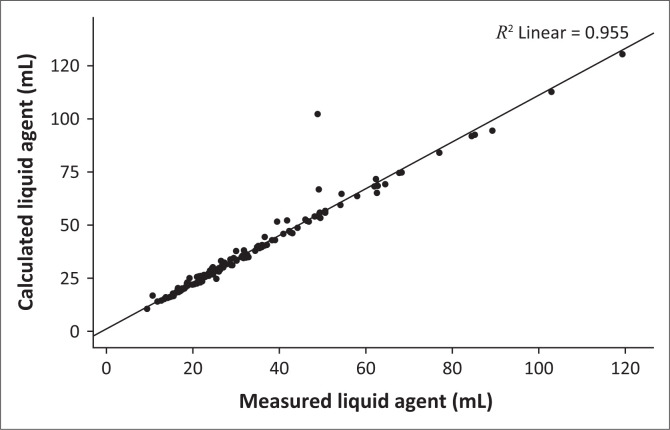
Linear correlation of calculated versus measured liquid agent consumption.

**FIGURE 4:**
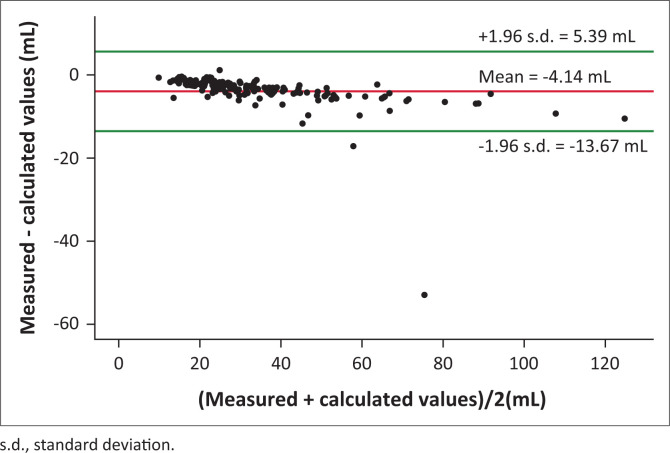
Bland-Altman analysis of calculated versus measured liquid agent consumption.

The average calculated LAC for induction, maintenance and case total are shown in Table 2. One-way ANOVA test showed no significant difference in total calculated LAC across theatres, F (9, 127) = 1.58, *p* = 0.127; therefore results were pooled. The Pearson correlation in Figure 3 showed a significant correlation between total calculated and measured LAC, *r* = 0.977, *p* < 0.001. The regression model showed that measured LAC explained 95.5% (*R*^2^ = 0.955) of the variance in calculated LAC, F (1, 135) = 2865.63, *p* < 0.001. Measured LAC was a significant predictor of calculated LAC, β = 1.099, *p* < 0.001. In the Bland-Altman analysis of pairs shown in Figure 4, 135 (98.5%) of the values were within the ± 1.96 standard deviation (s.d.). The average measured LAC was 32.69 ± 18.82 mL, whereas the calculated consumption showed a systematic average overestimation by 4.14 ± 4.86 mL (12.98 ± 10.41%).

**TABLE 2:** Liquid agent consumption and expenditure (*N* = 137).

Variable	Sevoflurane (mean ± s.d.)
Calculated Induction LAC (mL)	7.74 ± 6.91
Calculated Maintenance LAC (mL)	28.01 ± 20.43
Total Calculated LAC (mL)	36.84 ± 21.17
Measured LAC (mL)	32.69 ± 18.82
Difference to control (mL)	4.14 ± 4.86
Difference to control (%)	12.98 ± 10.41
LAC duration (min)	145.76 ± 95.42
Calculated induction LAC over time (mL/min)	1.15 ± 0.51
Calculated maintenance LAC over time (mL/min)	0.21 ± 0.1
Calculated case average LAC over time (mL/h)	16.71 ± 7.25
Calculated average case cost (ZAR)	119.72 ± 68.79
Calculated average case cost over time (ZAR/h)	54.32 ± 23.55

LAC, liquid agent consumption; ZAR, South African rand.

The total calculated LAC across all theatres was 5046.49 mL, with 1060.07 mL in induction and 3837.08 mL in maintenance. This was higher than the measured LAC of 4479.10 mL. While the induction phase is relatively short (4.6% of the total), its LAC was substantial, accounting for 21% of the calculated LAC. The cost of sevoflurane was determined using the 2023 government procurement cost of ZAR813.46 per 250 mL bottle, or approximately ZAR3.25/mL. Across all theatres during the study period, the total expenditures for calculated LAC, induction, maintenance and measured LAC were ZAR16401.10, ZAR3445.23, ZAR12470.51, and ZAR14 557.00, respectively.

The case average FGF had a very high positive correlation with the calculated cost of sevoflurane, *r* = 0.86, *p* < 0.001. The line of best-fit equation was mL of sevoflurane per minute = 0.02 + 0.14*x. The regression model showed that the case average FGF explained 74.15% of the variance in calculated LAC over time, which is, therefore, expenditure. The ANOVA test showed that the regression model was statistically significant, F (1,135) = 387.29, *p* < 0.001, *R*^2^ = 0.741. Another factor that had a high positive correlation to calculated expenditure was the maintenance FGF, *r* = 0.65. Factors such as induction FGF, maintenance ETS and MAC had no significant or low positive correlation with the calculated sevoflurane expenditure.

## Discussion

Inhalational anaesthetic gases exert their physiological effects in proportion to their partial pressure, which remains constant at various altitudes.^22^ Despite measuring partial pressure, gas analysers are calibrated in percentages, which can lead to dosing errors.^23^ While vaporiser output remains constant, measured concentration increases with decreasing barometric pressure.^23^ According to James,^22^ the recommended ETS concentration at an altitude of 1524 m is 2.4%, which is the same average value obtained in our study. This shows that the appropriate depth of anaesthesia was maintained. Paediatric theatres (1 and 2) had higher ETS concentrations as expected because younger patients typically require higher concentrations than adults.^24^ The average maintenance MAC was 0.90 ± 0.10 which had been calibrated for altitude. The accuracy of this MAC value relied heavily on anaesthetists entering the correct patient age, a task performed inconsistently, thus raising concerns about its reliability in our study.

Similar to findings by Kennedy et al.,^16^ published literature often excluded the initial high FGF period associated with the induction phase of anaesthesia. Our study included this initial period to obtain a comprehensive assessment of sevoflurane consumption. The significance is seen with the case average FGF (1.79 L/min) being statistically significantly higher than the average maintenance FGF (1.41 L/min), *p* < 0.001. Our time-weighted average FGF was 1.73 L/min. This value was significantly higher than the case average FGF published by Kennedy et al.^16^ during various phases of their study. Our study’s higher case average FGF was due to higher induction FGF and the induction period being more than twice as long. Our average maintenance FGF was also more than double those reported in their study.

Leijonhufvud et al.^25^ found that induction FGF of 4 L/min at 3 MAC vaporiser setting (6%) was optimal in increasing VA concentration from 0 to 1 MAC value within a minute. Although Leijonhufvud et al.^25^ used a test lung, their findings that time to reach 1 MAC plateaued at FGF of 4 L/min – 5 L/min were significant and supported work performed by Mapleson.^26^ A 1-1-8 wash-in scheme proposed by Tribuddharat et al.^27^ showed that the initial high FGF period might not be necessary as VA concentrations of 1% to 3.5% used in daily practice can be achieved within 5 min using total FGF of just 2 L/min. With our average induction FGF of 7.07 L/min, the first strategy in achieving LFA can be to encourage our anaesthetists to lower this value by using an optimal FGF of 4 L/min. This would be equivalent to approximately 43% reduction in FGF with significant potential cost savings and reduction in environmental pollution. The 25th centile for induction FGF was 6 L/min. With the default set FGF of 6 L/min, most of our anaesthetists increase this FGF to preoxygenate patients but fail to lower FGF once VA is switched on.

The induction duration of the 50th and 75th centiles in our study were 6 min and 9 min, respectively. A significant proportion of anaesthetists kept FGF high longer than required. Our induction durations are in stark contrast to those by Kennedy et al.,^16^ whereby 75% of their cases had induction times less than 4 min. They also suggested induction durations of 3 min – 5 min as a reasonable and attainable target. Implementing LFA cost-containment and awareness programmes is therefore imperative as there is a need to reduce induction FGF and duration at our institution.

Because of the lack of an integrated anaesthesia electronic record-keeping solution, work done by Biro^19^ to estimate our LACs using VA concentration and FGF was used. A very high positive correlation existed between our calculated and measured values (Figure 3). Our calculated LAC was overestimated by 4.14 ± 4.86 mL, representing 12.98 ± 10.41% (Figure 4). Overall, 98.5% of the calculated LAC values were within ± 1.96 SDs of the measured value. This is similar to findings by Biro et al.,^28^ showing agreement between our calculated and measured values. However, unlike in their study, our calculations overestimated rather than underestimated the true values.

The calculated average LAC was 16.71 ± 7.25 mL/h, which equates to ZAR54.32 ± 23.55/h. Using our model, sevoflurane (mL/min) = 0.02 + 0.14 × FGF (L/min), at case average FGF of 0.75–1 L/min, we predict an hourly sevoflurane consumption of 7.5 mL/h – 9.6 mL/h, respectively. Efforts to reduce the current time-weighted average FGF from 1.73 L/min to 0.75 L/min – 1 L/min could potentially yield a 39% – 52% reduction in the hourly consumption of sevoflurane. With extensive education and behavioural changes, these savings could be higher as our study excluded open circuit cases where FGF and LAC tend to be significantly higher.

In our hospital, we estimate that 12 600 volatile anaesthesia cases are performed annually, with an average case duration of 140 min. The current annual pharmacy spending on sevoflurane is ZAR1 541 412.00 or approximately 474 280 mL of sevoflurane. A 39% – 52% savings would potentially reduce annual expenditure by ZAR601 150.00 – ZAR801 534.00 ($31 600.00 – $42 200.00). Using equations by Edmonds et al.^29^ we estimate that our hospital could reduce pollution by 15 798 kg – 21 065 kg carbon dioxide equivalent (CO_2_e) sevoflurane annually. The potential cost savings and reduced environmental pollution warrant urgent implementation of LFA practice in our hospital.

This study has determined the conduct of inhalational anaesthesia in a South African tertiary hospital and successfully identified potential cost savings and waste reduction areas. Despite being a single-centre study, this does not limit the generalisability of its findings, which shows a need to teach the principles of LFA. Potential study limitations include the unknown Maquet^®^ vaporiser performance at different FGF rates.^30^ However, the vaporisers’ electronic injection system ensures accurate measurement of liquid agent quantities, with a specified accuracy of ±15% of the set value or ±5% of the maximum user setting, whichever is greater.^21^ This might explain the discrepancy in our calculated versus measured LAC values. While directly weighing the vaporiser offered a more accurate approach, implementing it within the study would have resulted in insurmountable logistical obstacles, ultimately rendering it impractical. The 1-min data intervals limited the accuracy of the results; however, the impact on clinical interpretation is likely negligible. Another postulated cause for overestimation was the lack of a well-calibrated barometer in theatre. However, this effect in our calculations was minimal as we found that significant changes in the reported barometric pressures yielded minimal change (< 0.5 mL) in the calculated LAC. Theatre temperature variations were also found to have insignificant changes to our calculated consumption. Despite these limitations, our approach is reliable and suitable for pharmacoeconomic estimations.

## Conclusion

In this study, we determined FGF at various phases of anaesthesia, VA concentration and MAC across multiple theatres in our hospital. We have demonstrated a reliable predictive model for LAC, with case average FGF identified as the primary determinant of overall consumption. On average, anaesthetists at our institution were found to be using medium-flow anaesthesia. Through our model, we can reasonably predict potential cost savings and pollution reduction when lower case average flows are targeted. The current climate emergency demands immediate, sustainable and comprehensive efforts to reduce greenhouse gas emissions across all sectors of society to avert its devastating effects on human health, food security and the biosphere.^31^ The financial benefits of LFA, coupled with its reduced environmental impact, allow anaesthesiologists to also play an essential role in reducing the effects of climate change.
